# Synthetic ligands of death receptor 5 display a cell-selective agonistic effect at different oligomerization levels

**DOI:** 10.18632/oncotarget.10508

**Published:** 2016-07-09

**Authors:** Julien Beyrath, Neila Chekkat, Cristian R. Smulski, Caterina M. Lombardo, Marie-Charlotte Lechner, Cendrine Seguin, Marion Decossas, Maria Vittoria Spanedda, Benoît Frisch, Gilles Guichard, Sylvie Fournel

**Affiliations:** ^1^ Institut de Biologie Moléculaire et Cellulaire, UMR 3572, Laboratoire d'Immunopathologie et Chimie Thérapeutique, Strasbourg 67084, France; ^2^ Institut Européen de Chimie et Biologie, UMR 5248, Institut de Chimie & Biologie des Membranes & des Nano-objets (CBMN), Univ. Bordeaux, Pessac 33607, France; ^3^ UMR 5248, CBMN, CNRS, Pessac 33600, France; ^4^ Current address: Faculté de Pharmacie, UMR 7199, Laboratoire de Conception et Application de Molécules Bioactives, Illkirch BP 67401, France; ^5^ Current address: Khondrion BV, Nijmegen 6525EX, The Netherlands; ^6^ Current address: University Medical Center Freiburg, Center for Chronic Immunodeficiency, Freiburg D-79110, Germany; ^7^ UMR 5248, CBMN, Univ. Bordeaux, Pessac 33600, France

**Keywords:** apoptosis, DR5, oligomerization, peptides, agonist

## Abstract

DR4 (Death Receptor 4) and DR5 (Death Receptor 5) are two potential targets for cancer therapy due to their ability to trigger apoptosis of cancer cells, but not normal ones, when activated by their cognate ligand TRAIL (TNF related apoptosis-inducing ligand). Therapies based on soluble recombinant TRAIL or agonist antibodies directed against one of the receptors are currently under clinical trials. However, TRAIL-R positive tumor cells are frequently resistant to TRAIL induced apoptosis. The precise mechanisms of this resistance are still not entirely understood. We have previously reported on synthetic peptides that bind to DR5 (TRAIL^mim/DR5^) and induce tumor cell apoptosis *in vitro* and *in vivo*. Here, we showed that while hexameric soluble TRAIL is able to efficiently kill the DR5 positive lymphoma Jurkat or the carcinoma HCT116, these cells are resistant to apoptosis induced by the divalent form of TRAIL^mim/DR5^ and are poorly sensitive to apoptosis induced by an anti-DR5 agonist monoclonal antibody. This resistance can be restored by the cross-linking of anti-DR5 agonist antibody but not by the cross-linking of the divalent form of TRAIL^mim/DR5^. Interestingly, the divalent form of TRAIL^mim/DR5^ that induced apoptosis of DR5 positive BJAB cells, acts as an inhibitor of TRAIL-induced apoptosis on Jurkat and HCT116 cells. The rapid internalization of DR5 observed when treated with divalent form of TRAIL^mim/DR5^ could explain the antagonist activity of the ligand on Jurkat and HCT116 cells but also highlights the independence of the mechanisms responsible for internalization and activation when triggering the DR5 apoptotic cascade.

## INTRODUCTION

Tumor necrosis factor (TNF)-related apoptosis-inducing ligand (TRAIL/Apo2L/TNFSF10) belongs to the TNF ligand superfamily [1] and triggers apoptosis. TRAIL bears great value for cancer therapy as it preferentially kills tumor cells while sparing normal ones from death [2, 3]. There are five different TRAIL receptors: two fully functional called DR4 (TNFRSF10A/DR4) and DR5 (TNFRSF10B/R5), two cell-bound receptors unable of transmitting an apoptotic signal, TRAIL-R3 (TNFRSF10C/DcR1) and TRAIL-R4 (TNFRSF10D/DcR2), and a soluble receptor called osteoprotegerin (OPG) that also interacts with the RANK ligand [4]. Triggering of DR4 and DR5 results in formation of a death inducing signaling complex (DISC) leading to caspase activation and apoptosis [5].

The unique characteristic of TRAIL encouraged the development of TRAIL-R agonists, including recombinant TRAIL protein and monoclonal antibodies targeting specifically DR4 or DR5, which have demonstrated efficient anticancer activities in a number of preclinical studies [6]. However, results from clinical trials showed that although TRAIL agonists have shown low toxicity in patients, only weak therapeutic effect was observed when used in monotherapy [7]. It has emerged that some cancer cells, especially primary cancer cells, acquired resistance to TRAIL-induced apoptosis. Multiple mechanisms for TRAIL resistance that can affect many components of the TRAIL signaling pathways have been identified [8]. Major mechanisms include overexpression of anti-apoptotic molecules like c-FLIP as well as overexpression of proteins belonging to the Bcl-2 family or Inhibitory of Apoptosis Protein (IAP) family. In some cases, TRAIL-R agonists induced activation of non-cell death signaling pathways such as NFkB, MAPK, Scr, and phosphoinositide 3-kinase (reviewed in [9]). In addition, mutations could generate nonfunctional DR4/5 receptors [10, 11]. Finally, resistance can be acquired following an inefficient receptor clustering at the membrane level, thus limiting DISC component recruitment [12]. To overpass these resistances, it is crucial to better characterize the mechanisms of apoptosis induction including early events occurring at the membrane level.

TRAIL is a type II transmembrane protein, active in a membrane-bound form or as a soluble cytokine after proteolytic shedding [13, 14]. The C-terminal extracellular domain forms homotrimers that mediate receptor binding. Although this ligand-induced oligomerization is generally accepted to generate the minimal active unit, some members of the family require a higher degree of oligomerization to trigger effective signaling [15, 16]. Using a soluble FLAG-tagged form of TRAIL (sTRAIL) enabling its cross-linking, Wajant H *et al* [17] concluded that DR4 was activated by both cross-linked and non-cross-linked sTRAIL whereas DR5 required cross-linked sTRAIL, and to a larger extent a membrane-bound TRAIL. This difference in receptor oligomerization between DR4 and DR5 required for apoptosis induction has also been reported with specific antibodies directed against each receptor on chronic lymphocytic leukemia, where cross-linking of anti-DR5 *via* a secondary antibody was necessary to efficiently kill the cells [18].

However, all these observations might certainly be cell and agonist specific. Indeed, several groups have reported the apoptogenic activity of non-cross-linked antibodies directed against DR5 [19–21]. We previously reported the activity of divalent peptidic agonists of the DR5 receptor (named as TRAIL^mim/DR5^) [22, 23] and the functional impact of the multimerization by using adamantane-based dendrons [24]. Finally, the work from Thomas and coworkers [25], clearly illustrates some mechanistic specificities in the triggering of DR5 pathway by various agonists, highlighting the fact that activation of TRAIL-Rs is agonist specific and might not only depend on oligomerization and therefore bringing this system to a higher level of complexity. Moreover, little is known about the TRAIL receptor internalization requirement in TRAIL induced apoptosis. It has been proposed that TRAIL ligation induces rapid TRAIL-R internalization primarily by clathrin-dependent endocytosis but also by clathrin-independent endocytosis. However, the involvement of this receptor internalization in apoptotic signaling is still controversial [26–28].

Here, we used HCT116, BJAB and Jurkat cells to further characterize our peptidic ligands and gain insight on the DR5 activation mechanisms. The present study demonstrates that while the three cell lines are sensitive to a cross-linked form of TRAIL, Jurkat and HCT116 cells are resistant to apoptosis induced by the divalent form of TRAIL^mim/DR5^ as well as by an anti-DR5 agonist monoclonal antibody. Moreover, caspase-8 is not recruited to DR5 upon treatment of Jurkat cells with TRAIL^mim/DR5^. The resistance of Jurkat and HCT116 cells was overcome by the cross-linking of anti-DR5 antibody but not by cross-linking of the divalent form of TRAIL^mim/DR5^.

Furthermore, we show that divalent TRAIL^mim/DR5^ can specifically inhibit apoptosis induced by the cross-linked form of TRAIL, thus acting as an antagonist. More surprisingly, divalent TRAIL^mim/DR5^ induced a rapid internalization of DR5 in HCT116, BJAB and Jurkat cells, a phenomenon explaining its antagonist activity. In summary we show that divalent TRAIL^mim/DR5^ selectively bind to DR5 and induce its internalization in BJAB, HCT116 and Jurkat cells, but could only induce DISC formation, and by so, the apoptotic machinery activation, in BJAB cells.

## RESULTS

### Divalent TRAIL^mim/DR5^ induces apoptosis of BJAB cells but not Jurkat or HCT116 cells

**Figure 1:**
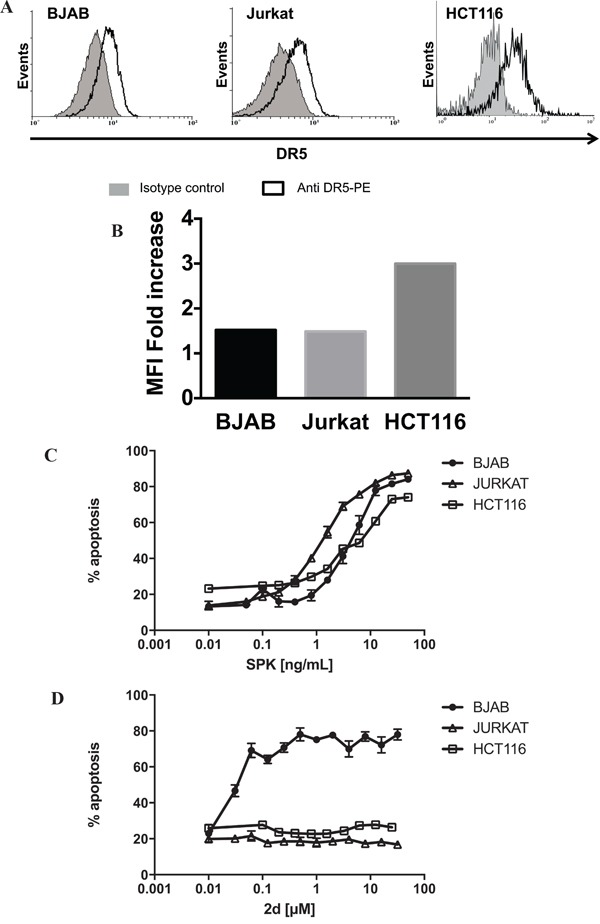
Differential apoptogenic activity of SPK and TRAIL^mim/DR5^ BJAB Jurkat and HCT116 cells **A.** were stained with monoclonal antibody directed against DR5 or a control isotype coupled to phycoerythrin (PE) and analyzed by flow cytometry. Results in **B.** showed fold increase of the mean of fluorescence intensity between control isotype and TRAIL-R2 specific labeling that was calculated to compare level of TRAIL-R2 expression on the three cells lines. BJAB, Jurkat and HCT116 cells were treated with stepwise 2-fold increasing concentrations of SPK **C.** or divalent TRAIL^mim/DR5^ peptide (2d) **D.** After 16 hours of incubation, apoptosis was determined as the percentage of Annexin V–positive cells by flow cytometry analysis. Results are expressed as mean % apoptosis of 3 independent experiments +/− SD.

DR5 is known to require a high degree of oligomerization in order to activate the apoptotic machinery [29] and as expected, the pro-apoptotic DR5-specific peptides we developed (TRAIL^mim/DR5^) are active only as divalent or trivalent forms [22]. Divalent TRAIL^mim/DR5^ display great therapeutic potential as shown by their ability to selectively induce a DR5-dependent apoptosis in cancer cells *in vitro* and by their tumoricidal activity *in vivo* [30]. To further characterize the mode of activation of divalent TRAIL^mim/DR5^, we compared the activity of one member in this series (referred here to as 2d, see supporting information for corresponding formula) on the B cell lymphoma BJAB, the T cell lymphoma Jurkat and the epithelial colorectal carcinoma HCT116 that show comparable DR5 surface expression (Figure 1A and 1B) in comparison with the activity of the human recombinant (rh) hexameric form of TRAIL (SuperKiller TRAIL, referred to as SPK). Cells were incubated with stepwise 2-fold increasing concentrations of SPK or 2d for 16 hours and percentage of apoptosis was measured by detection of phosphatidylserine externalization after co-labeling with Annexin V-FITC/propidium iodide. Whereas SPK induced apoptosis in a dose dependent manner of HCT116, BJAB and Jurkat cells, with more than seventy percent of apoptosis at doses over 10ng/mL (Figure 1C), 2d peptide induced apoptosis of only BJAB cells (Figure 1D). SPK EC50s were about 1.5ng/mL on Jurkat and 5ng/mL on BJAB or HCT116 cells, conferring a 3.3 fold difference only between BJAB and Jurkat. By contrast, 2d peptide EC50 was 0.05μM (Figure 1D) on BJAB cells but no induction of apoptosis was observed when the Jurkat or HCT116 cell lines were treated with 2d peptide up to 32μM, a concentration 600 times higher than the EC50 on BJAB cells. Taken together, the results suggest that induction of apoptosis on Jurkat and HCT116 cells by our peptide may require an extended oligomerization state of DR5.

### BJAB, HCT116 and Jurkat cells require different DR5 receptor oligomerization for apoptosis induction

**Figure 2:**
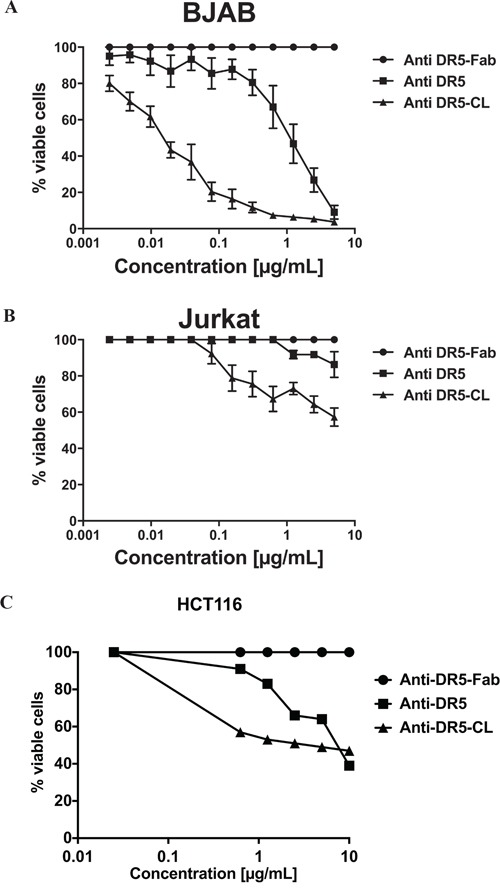
Different requirement of DR5 oligomerization for apoptosis induction BJAB **A.**, Jurkat **B.** and HCT116 **C.** cells were treated with an agonist antibody directed against DR5 processed for different levels of valencies: monovalent (anti-DR5-Fab), divalent (anti-DR5), and tetravalent (anti-DR5-CL). After 16 hours of incubation cell viability was determined using the MTS assay. Results are expressed as % of cell viability inhibition according to the following formula viable cell % =(OD (treatment) / OD (100% viability) * 100) were cells incubated with medium alone were considered as 100% of viability. Results are expressed as the OD mean of 3 independent experiments ± SEM.

To emphasize the potential implication of ligand-induced DR5 oligomerization in the discrepancy between BJAB on one side and Jurkat and HCT116 cells on the other side, they were treated with a specific anti-DR5 agonist antibody in different state of oligomerization, i.e. monovalent Fab fragment (anti-DR5-Fab), complete divalent antibody (anti-DR5) and cross-linked antibodies (anti-DR5-CL; partially tetravalent form). As expected, anti-DR5-Fab did not induce cell death in the three cell lines. The complete divalent anti-DR5 induced a high level of apoptosis only in BJAB cells with 80% of dead cells at 5μg/mL. By contrast, it induced only a slight level of apoptosis of Jurkat cells with only 10% of dead cells at 5μg/mL and a middle one on HCT116 with approximately 36% at 5μg/mL (Figure 2).

Interestingly, the anti-DR5-CL increased apoptosis induction, in comparison to anti-DR5, on BJAB and HCT116 cells (Figure 2A and 2C), but more importantly, it induced apoptosis on Jurkat cells (Figure 2B) up to 40% of dead cells at 5μg/mL. Noticed that the anti-DR5-CL induced apoposis on Jurkat and HCT116 cells in a lesser extend than in BJAB cells which reach almost 100% of apoptosis at less than 1μg/mL.

These results suggest that the different sensibility of BJAB, HCT116 and Jurkat cells in 2d-induced apoptosis could be explained by the fact that the degree of oligomerization of DR5 required to induce cell death is different between the three cell lines, Jurkat and, in a lesser extend, HCT116 requiring a higher oligomerization state. Moreover we can hypothesize that multivalent ligands such as hexameric ligands might increase DR5-induced apoptosis in Jurkat and HCT116 cells.

### DR5 induced apoptosis on Jurkat cells require recruitment of more than two DR5 receptors

**Figure 3:**
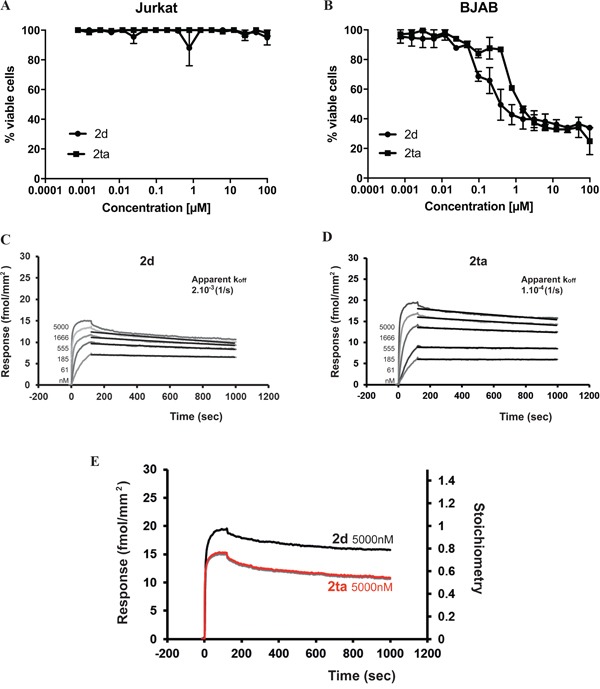
Tetravalent version of TRAIL^mim/DR5^ peptide is not sufficient for triggering Jurkat cell death Jurkat **A.** or BJAB **B.** cells were treated with the divalent TRAIL^mim/DR5^ peptide (2d) and the tetravalent version (2ta). After 16 hours of incubation, cell viability was determined using the MTS assay as described in Figure 2. Kinetic curves of divalent and tetravalent TRAIL^mim/DR5^ peptides binding to immobilized human TRAIL-R2 (density of TRAIL-R2 was 65 fmol/mm^2^) were obtained by SPR experiments (**C**, **D**). Peptides 2d and 2ta were tested at concentrations of 5000, 1666, 555, 185, 61 nM. The response is expressed in fmol/mm^2^ (1000RU= 1mg/mm^2^). The apparent k_off_ were evaluated by fitting (black curves) the post-injection phase of the curves and values are the mean of three experiments. **E.** Responses obtained at 5μM were normalized to the stoichiometry of interaction.

To go further in the understanding of DR5 oligomerization requirement for efficient apoptosis signaling in Jurkat cells, we developed a tetravalent version of TRAIL^mim/DR5^ (referred here to as 2ta). For that, we first prepared a divalent TRAIL^mim/DR5^ with a specific trifunctionalized linker, which was subsequently engaged in a dimerization reaction to give 2ta. Unlike cross-linked antibodies whose valency is not precisely defined, 2ta is chemically characterized as a tetramer. All experimental details related to the preparation of the new linker and 2ta as well as analytical data can be found in supporting information. We then compared the efficiency of 2ta and 2d to induce cell death in Jurkat and BJAB cells. No apoptosis induction was observed when Jurkat cells were treated with 2ta peptide (Figure 3A). Unexpectedly, in BJAB cells, 2ta peptide triggered cell death at the same extent than 2d peptide (Figure 3B). To understand the absence of activity gain with 2ta on the two cell lines, we explored the ability of 2ta peptide to bind to human DR5 using Surface Plasmon Resonance (SPR). Recombinant human DR5 was immobilized on a sensor surface and the 2d or 2ta peptides were flushed at different concentrations over empty and DR5 surfaces. As the model of interaction was complex, we only evaluated an apparent k_off_ from the post-injection phases using the Langmuir binding model (Figure 3C and 3D). Interestingly, results showed equivalent apparent k_off_ for 2ta and 2d indicating that peptide tetramerization did not enhance the stability of the ligand/receptor complexes. More surprisingly, when we normalized the maximal response (ie 5μM) obtained for each peptide to the stoichiometry of interaction between peptides and DR5, we observed that 2d peptide engaged two DR5 receptors (stoichiometry = 0.5) whereas the 2ta peptide recruited only one DR5 receptor (stoichiometry = 1) (Figure 3E) probably due to the steric distribution of peptide subunits in the oligomer. Taken together, valence and spatial distribution seems to be two determining factors, most probably distinct for different cell lines, in order to efficiently trigger apoptosis in each target cell.

Divalent TRAIL^mim/DR5^ inhibits SPK-induced apoptosis of Jurkat cells by inducing DR5 internalization.

**Figure 4:**
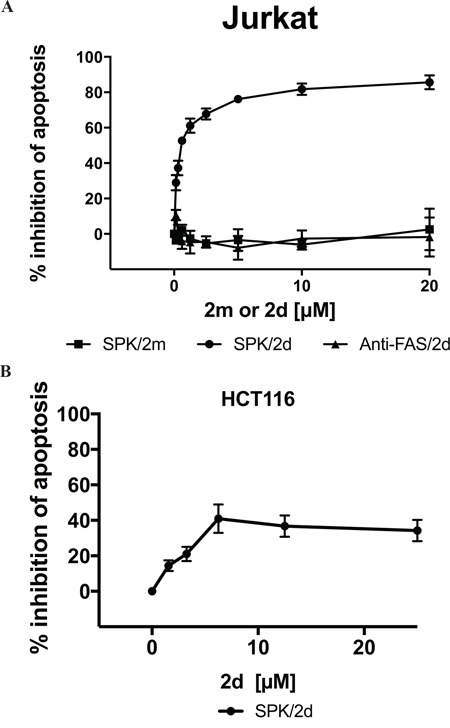
Divalent TRAIL^mim/DR5^ inhibits SPK mediated Jurkat and HCT116 cells apoptosis Jurkat **A.** or HCT116 **B.** cells were treated with a solution consisting of a constant concentration of SPK (5ng/mL) or an agonist antibody (5ng/mL) directed against Fas and stepwise 2-fold increasing concentration of divalent (2d) or monovalent (2m) TRAIL^mim/DR5^ peptides. After 16h of incubation, apoptosis was determined as the percentage of Annexin V–positive cells by flow cytometry. Data are expressed as the percentage of inhibition of apoptosis.

The absence of 2d or 2ta efficiency in inducing Jurkat cell apoptosis does not permit to exclude the possibility that the TRAIL^mim/DR5^ fail to engage DR5 at the surface of Jurkat cells. Using SPR, we previously reported that both monovalent and divalent forms of TRAIL^mim/DR5^ competed with rhTRAIL for binding to rhDR5 in a concentration-dependent manner, with a 10-fold difference of the IC50 [30]. Thus, we tested the ability of TRAIL^mim/DR5^ to compete with SPK in apoptosis induction. For that, cells were incubated for 16 hours with of a constant concentration of SPK (5ng/mL) and stepwise 2-fold increasing concentrations of peptide 2m (monovalent) or 2d (divalent) and apoptosis was determined by detection of phosphatidylserine externalization after co-labeling with Annexin V-FITC/propidium iodide. At this concentration of SPK, 45 % of Jurkat cells became apoptotic. Co-incubation with 2d, but not with 2m, inhibited the apoptosis induction mediated by SPK in a dose dependent manner with a 2d IC50 of 0.3μM (Figure 4A). Moreover, the inhibition observed is restricted to DR5 since 2d peptides could not inhibit apoptosis induced by an agonist antibody (CH11) directed against Fas, another pro-apoptotic member of the TNFR superfamilly (Figure 4A). An inhibition was also observed in HCT116 cells but in a lesser extend (40% of inhibition at 20μM of 2d). Altogether, these results suggest that TRAIL^mim/DR5^ binds to DR5 and that divalent structure of the peptide is required for effective inhibition of SPK induced cell death.

**Figure 5:**
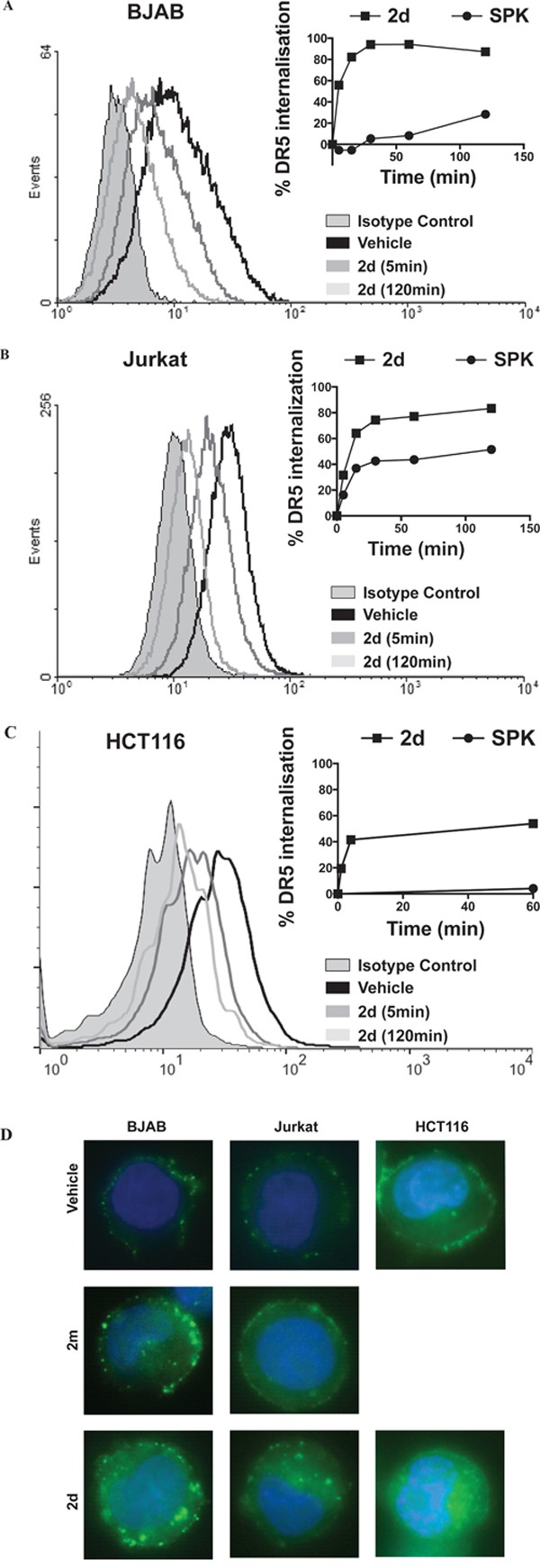
SPK and divalent TRAIL^mim/DR5^ induce DR5 internalization on BJAB Jurkat and HCT116 cell lines BJAB **A.**, Jurkat **B.** and HCT116 **C.** cells were treated with SPK or divalent (2d) TRAIL^mim/DR5^ peptide for different period of times. Cells were subsequently processed for surface expression of DR5 analysis by flow cytometry. The panels show histograms for selected times during a kinetic analysis of DR5 internalization after treatment with divalent (2d) TRAIL^mim/DR5^. The right panels represent the percentage of DR5 internalization upon treatments calculated using the mean fluorescence intensity values at each time point. D. BJAB, Jurkat or HCT116 cells were treated for 30 minutes with vehicle, monovalent (2m) or divalent (2d) TRAIL^mim/DR5^ peptides. Cells were subsequently processed for either surface expression of DR5 analysis by localization of DR5 by immunocytochemistry. Results showed representative images of DR5 localization in the three cell lines.

It was described that DR5 endocytosis occurs after TRAIL treatment in BJAB cells, although it was not required, in this cell line, for apoptosis pathway activation [26]. To determine whether TRAIL^mim/DR5^ induced DR5 endocytosis in BJAB cells but also in HCT116 and Jurkat cell lines, cells were incubated with a dose (EC50) of 2d or SPK for different time periods and assessed for DR5 surface expression by flow cytometry. In accordance with the litterature, SPK induced internalization of DR5 on the BJAB cells, with a maximum of 30 % after 2 hours of incubation in our experimental conditions. 2d had the same effect, although with faster kinetic since DR5 internalization reached almost 95% after one hour of incubation (Figure 5A). More surprisingly, the same profiles were obtained with both SPK and 2d when incubated with Jurkat cells (Figure 5B). Indeed, the kinetic of internalization of DR5, reaching 65 % after 15 minutes and a maximum of 85 % after 2 hours upon 2d treatment, was faster than upon SPK treatment, reaching only 37 % after 15 minutes and 52 % after 2 hours. In HCT116, 2d induced a clear internalization of DR5 (approximately 50% after 1 hour) whereas SPK did not induce any internalization. In all cases, internalization of DR5 was confirmed by immunocytochemistry experiments. A clear cytosolic localization of DR5 was depicted in BJAB, HCT116 and Jurkat cells treated with 2d while it localized at the plasma membrane in the non-treated cells or cells incubated 30min with 2m (Figure 5D). According to the fast kinetic of DR5 internalization following 2d incubation, this phenomenon might explain the desensitization of Jurkat cells and partial desensitization of HCT116 cells to SPK, rather than a competition for a binding site. It is also in accordance with results obtained with the monovalent form of TRAIL^mim/DR5^ that specifically binds DR5, did not induced DR5 internalization and did not inhibited SPK-induced Jurkat cell apoptosis.

Taken together, these results show that divalent TRAIL^mim/DR5^ can induce rapid internalization of DR5 in BJAB, HCT116 and Jurkat cells but can trigger apoptosis only in BJAB and not in HCT116 or Jurkat cells, acting in one case as an agonist and in another one as an antagonist.

## DISCUSSION

Since its discovery in the mid 90′s [31, 32], TRAIL rapidly appeared as the promising cytokine in the family of cancer therapeutics, as it selectively induces apoptosis of tumor cells while sparing normal ones from death. Although the physiological role of TRAIL is not totally elucidated, different strategies to exploit this pathway for cancer therapy are currently under clinical investigation [6, 30]. Indeed, recombinant TRAIL or activating antibodies directed against either DR4 or DR5, alone or in combination with other cytotoxic agents, have reached phase II in clinical trials for a diverse set of cancers. Other potential approaches have been reported such as the adenoviral delivery of TRAIL coding sequence into tumor cells [33–34] or the more “drug-like” synthetic multivalent ligands of DR5 (TRAIL^mim/DR5^)[22].

However, targeting TRAIL-Rs in cancer as monotherapy is limited by acquired resistance in many cancer cells. The major cause of resistance described in the literature refers to the overexpression of anti-apoptotic proteins. Indeed, c-FLIP is a crucial regulator that inhibits the pro-apoptotic activity of the DISC. Overexpression of c-FLIP has been linked to TRAIL resistance in many cancers [35] and downregulation of c-FLIP using synthetic inhibitors enhances TRAIL-induced apoptosis [36]. Moreover the anti-apoptotic members of the Bcl-2 family proteins inhibit apoptosis by influencing the permeability of the mitochondrial membrane. Overexpression of Bcl-2, Bcl-XL and Mcl-1 has been reported to result in TRAIL resistance in mitochondria-dependent type II cancer cells [37, 38]. IAPs are regulator of apoptosis by modulating caspase activity. The most studied IAPs, the X-linked inhibitors of apoptosis (XIAP), was for instance shown to mediate TRAIL resistance when overexpressed [39]. In addition to these intracellular resistances, resistance in TRAIL induced apoptosis at the membrane level is not clearly studied yet. In this regard, the main resistance described is the apparition of mutations in DR4 or DR5 leading to dysfunctional receptors or the decrease of DR5 or DR4 expression correlated for example to mutations in Ras oncogene [40–41].

At a molecular level TRAIL is organized as a homotrimer, as other members of the TNF-superfamily, a structural feature accepted to be the minimal unit required to trigger receptor signaling. Indeed, it was shown that the membrane-bound, but not soluble form of TNF-α, is the prime activating ligand for TNF-R2 (TNFRSF1B), while TNF-R1 (TNFRSF1A) could be activated by both ligand forms [42, 43]. Fas (TNFRSF6) induced apoptosis is highly dependent on receptor oligomerization and requires at least two adjacent trimers for an effective signal [15]. However, some intrinsically divalent molecules such as antibodies or synthetic peptides are known to activate DR4- or DR5-dependent apoptosis. The reason why such molecules are able to trigger signaling through DR5 is not fully understood but can be rationally explained by the existence of pre-oligomerized form of the receptor at the surface of the cells [44] as it was also observed with other receptors of the family [45, 46]. In an opposite manner, it is generally accepted that extended oligomerization of recombinant TRAIL or antibodies against DR4 or DR5 can be required to exert maximal activity depending on the cell types.

Herein, we highlighted that DR5 induced apoptosis required different degree of oligomerization that can vary according to the cancer cells. Indeed, while divalent peptides or anti-DR5 antibody induced BJAB cell apoptosis, HCT116 and Jurkat cells required at least the DR5 antibody cross-linked (tetravalent ligand) to induce efficient apoptosis.

We developed a tetrameric version of TRAIL^mim/DR5^ peptide to recruit more DR5 receptors and thus increase DISC formation. Unfortunately, increasing the oligomerization state of TRAIL^mim/DR5^ did not enhance apoptosis in BJAB cells and did not induce apoptosis in Jurkat cells. This is surprising because antibody cross-linked led to apoptosis induction in Jurkat cells. In this condition, tetravalent peptide was not efficient certainly because of an inappropriate conformational presentation of TRAIL^mim/DR5^ peptides thus limiting the interactions between the four peptidic ligands and DR5 at the membrane level. In the same way, the DR5 specific hexameric peptide that we described in [24] did not induced Jurkat cells certainly because of a conformational issue. Thus, understanding the interaction between synthetic ligands and DR5 is crucial and will be helpful for the development of efficient multivalent ligands. Moreover, in the present study, we showed that poorly oligomerized synthetic ligands of DR5 could exert either an agonistic or an antagonistic activity in a cell-type dependent fashion. Up to date, this is the first demonstration of dual and opposite roles for ligands of DR5. The antagonist activity was explained by the fact that TRAIL^mim/DR5^ induced internalization of the receptor desensitizing the cells to recombinant TRAIL-induced apoptosis. As the TRAIL pathway is a natural component of the endogenous tumor-surveillance system in mammals [47–49], therapies based on poorly-oligomerized activators of TRAIL death receptors could potentially have negative effect on the tumor progression and dissemination.

The internalization of Fas and DR5 following the binding of their cognate ligands has already been reported [50], however, contrary to Fas, the role of DR5 internalization for induction of apoptosis is still controversial [26]. Here we show that the internalization of DR5 can arise without induction of apoptosis, confirming the independence of the two mechanisms. More interestingly, this mechanism was restricted to the divalent form of TRAIL^mim/DR5^ as the monovalent version of the peptide had no effect neither on the internalization nor the inhibition of recombinant TRAIL signaling.

Taken together, our results suggest that some ligands targeting the DR5 receptor have a cell dependent agonist or antagonist activity. Therefore, special care must be taken when considering DR5 as an anticancer target.

## MATERIALS AND METHODS

### Reagents

The Human recombinant hexameric ligand TRAIL named SuperKiller TRAIL (SPK) was obtained from Enzo life Sciences (Lausane, Switzerland). For apoptosis induction, agonist antibody against CD95/Fas (clone CH11) was purchased from Beckman coulter (Fullerton, USA). For immunoprecipitation analysis, anti-caspase-8 antibodies (clone C20) were purchased from Santa Cruz Biotechnology (California, USA) and anti-DR5 were from Chemicon (Temecula, CA). PE-labeled anti-DR5 and isotype control used in flow cytometry analysis were purchased from Diaclone (Besançon, France). Non-coupled anti-DR5 antibody (clone DJR2-4) and the secondary antibody from mouse, coupled to alexa-488 used for the immunocytochemistry experiment were obtained from ABDserotec (Düsseldorf, Germany) and Life technologies (Carlsbad, CA), respectively. TRAIL^mim/DR5^ peptides 2m, 2d and 2h were synthesized in our laboratories as previously described [22]. All details related to the preparation of 2ta are provided as supporting information.

### Cell lines

The Burkitt lymphoma BJAB were kindly provided by Andrew Thorburn (Department of Pharmacology, University of Colorado Denver School of Medicine, USA) and were maintained as previously described [24]. Jurkat cells were purchased from ATCC (American Type of Culture Collection, number TIB-152). The colorectal carcinoma HCT116 were purchased from ATCC (American Type of Culture Collection, number CCL247). Cells were cultured in RPMI 1640 supplemented with 10% FCS (Fetal Calf Serum) and gentamycin (10 μg/mL). Puromycin antibiotic (0.5μg/mL) was added to the BJAB medium to ensure the maintaining of DR5 expression. Media, FCS and antibiotics were from Sigma Aldrich (St Louis, MO, US).

### Apoptosis and cell viability measurements

BJAB and Jurkat cells (10^6^ cells/mL) were cultured in 100μL of culture medium in 96-well plates. The next day, cells were treated with the indicated concentrations of the different ligands. HCT116 (5.10^5^ cells/mL) were cultured and stimulated the same day in 100μL of culture medium in 96-well plates. Apoptosis was determined by detection of phosphatidylserine externalization after co-labeling with Annexin V-APC/propidium iodide, according to the manufacturer instructions or cell viability was evaluated by MTS assay.

For apoptosis determination, cells were analyzed by flow cytometry using a FACSCalibur flow cytometer (Becton Dickinson, Pont de Claix, France) and the data were analyzed using Flowjo software (Treestar, Ashland, USA). Apoptosis is displayed as the percentage of cells presenting an Annexin-V positive staining on SPK or synthetic peptides treatment. Inhibition of apoptosis was calculated as follows: 100-[% of Annexin V-positive cells X 100] / % of Annexin V-positive cells treated with rhTRAIL or anti-CD95/Fas.

MTS assay was performed according to the manufacturer specification (Promega Corporation, Madison, WI).

### Differential requirement of DR5 oligomerization analysis

For crosslinking assay, human IgG1 DR5 antibody was purchased from R&D systems (Clone 71903) and corresponds to the bivalent ligand. To obtain a monovalent ligand, DR5 antibody was digested with 0.25μg of papain (Sigma Aldrich) during 6h at RT in a digestion buffer consisting of 0.02M EDTA, 0.02M cysteine diluted in PBS. Digestion was stopped with addition of 0.3M Iodoacetamide. To obtain a tetravalent ligand, DR5 antibody was cross-linked by a goat anti mouse IgG, Fc fragment (Jackson ImmunoResearch) in a ratio 1:10 (v/v) at RT during 6h. To evaluate the impact of multivalent ligand on DR5 induced apoptosis, BJAB and Jurkat (10^6^ cells/mL) cells were treated with the different ligands and viability was measured by MTS assay as described above.

### Receptor internalization analysis

#### Flow cytometry

BJAB, HCT116 and Jurkat cells were treated with either SPK or 2d peptide for different time periods and washed once with cold PBS. Since that time cells were maintained at 4°C. DR5 was stained with PE-labeled anti-DR5 according to the manufacturer's instructions followed by cell fixation in a solution of 0.4 % paraformaldehyde. Cell surface expression of DR5 was analyzed by flow cytometry using a FACSCalibur flow cytometer, and the data were analyzed using Flowjo software for histogram representation. Percentage of internalization was calculated as follows: 100-[mean of fluorescence (MFI) X 100] / MFI of non-treated cells.

#### Immunocytochemistry

5×10^5^ BJAB, HCT116 or Jurkat cells were exposed to 2m (100nM) or 2d peptides (50nM) for 30 minutes at 37°C. Cells were then fixed with 2% paraformaldehyde during 15 minutes at room temperature, washed in TBS (Tris Buffered Saline) followed by incubation with primary antibody against DR5 (25μg/mL) (clone DJR2-4) in 0.2% saponin for 2 hours at room temperature. After two washing steps in TBS, the cells were incubated with the secondary antibody (2.5μg/mL) (polyclonal goat Ig F(ab')2 anti-mouse IgG(H+L)-alexa488) in 0.2% saponin for 30 minutes at 37°C. Cells were counterstained with the DNA dye DAPI (0.5 μg/mL) for 10 minutes at room temperature to identify nuclei, and images were collected by epifluorescence microscopy (Axiovert 200M, Zeiss, equipped with a Zeiss Apotome module) and processed by Adobe Photoshop CS2 software. The same procedure without the primary antibody step was run in parallel as a control for the specificity of the staining.

### Staining for flow cytometry analysis

Cells (10^6^) were washed in PBS containing 2% FCS and then incubated at 4°C for 20 min with the monoclonal antibody directed against DR5 or a control isotype both coupled to phycoerythrin (PE) (Diaclone, Besançon, France) used at a concentration recommended by the manufacturer. After two washes in PBS-2% FBS, cells were analyzed by flow cytometry (FACScalibur, Becton Dickinson).

### Surface plasmon resonance

Biosensor assays were performed on a Biacore T200™, at 25°C. The running buffer was HBS-EP buffer [10mM HEPES (pH 7.4) containing 0.15M NaCl, 3.4mM EDTA and 0.005% (v/v) Tween P20]. The human DR5 and RANK (Enzo Life Sciences, Farmingdale, NY) receptors were immobilized on a sCM5 sensor chip (GE Healthcare) using the standard amine coupling procedure. The receptors were diluted in 10mM acetate buffer (pH 5.0) at a concentration of 5μg/mL. The density of receptors immobilized on the sensor chip was 65 fmol/mm^2^ for DR5. Peptides were injected at a flow rate of 5μL/min for 120s and allowed to dissociate for an additional of 420s. Channels were then regenerated for 5s with 25mM HCl. All binding curves were double-referenced (i-e subtraction of the data of the empty flow cell followed by the subtraction of the data from a run buffer injection cycle). The Langmuir model was used to fit the post-injection phases of peptides (BIAevaluation version 4.1.1).

## CHEMISTRY EXPERIMENTAL FIGURES


