# Supplementary material for: Synthetic ligands of death receptor 5 display a cell-selective agonistic effect at different oligomerization levels
Source: Oncotarget. 2016 Jul 9;7(40):64942–56. doi: 10.18632/oncotarget.10508 (PMC5323128; doi:10.18632/oncotarget.10508)
Supplement: Supplementary file 1 [file oncotarget-07-64942-s001.pdf]

Synthetic ligands of death receptor 5 display a cell-selective agonistic effect at different oligomerization levels

CHEMISTRY EXPERIMENTAL

General

Resin, protected amino acids and coupling reagents were purchased from PolyPeptide Group (Strasbourg, France) and Iris Biotech (Marktredwitz, Germany). Chemical reagents were purchased from Sigma–Aldrich and Alfa-Aesar and peptide grade solvents were purchased from Carlo Erba. Analytic HPLC characterisations were performed on a Dionex Ultimate 3000 system, using a Macherey-Nagel column (Nucleodur cc 70/4 100-3 C18 ec, 4.6 x 100, solvents acetonitrile/water 0.1% TFA, 1 ml/min). Semipreparative HPLC was performed on a Dionex Ultimate 3000 system, using a Macherey-Nagel column (Nucleodur 100-16 C18 ec, 10 x 250, solvents acetonitrile/water 0.1% TFA, 4 ml/min). Preparative HPLC was performed on a Gilson Preparative HPLC system using a Macherey-Nagel column (Nucleodur C_{18} column 20×250 mm, solvents acetonitrile/water 0.1% TFA, 20 ml/min) and UV detection at 220 nm. Low resolution ESI mass spectra were recorded with an LCQ Advantage/LC Surveyor apparatus (Thermo Finnigan). High resolution ESI mass spectra were performed on a Thermo Exactive apparatus.

Synthesis of dimerisation linker (4)

N-Boc-4-((benzyloxy)carbonyl)-1-(2,2-dimethyl-4,6-dioxo-1,3-dioxan-5-yl)-2-amino-butane (1)

EDC.HCl (4.26 g, 22.2 mmol), DMAP (2.71 g, 22 mmol) and Meldrum's acid (2.13 g, 14,8 mmol) were added to a 0.25 M solution of Boc-Glu(OBn)-OH (5 g, 14.8 mmol) in CH_2Cl_2 at 0°C. The mixture was allowed to room temperature, stirred for 5 h, and then washed with 1N $KHSO_4$. The organic layer was dried over Na_2SO_4 and filtered prior to the addition of CH_2Cl_2 (to afford a 0.05 M solution) and 10 % v/v of AcOH. 1.67 g of $NaBH_4$ (3 eq, 44.4 mmol) was then added portionwise to the previous solution stirred at room temperature. After 12 h,

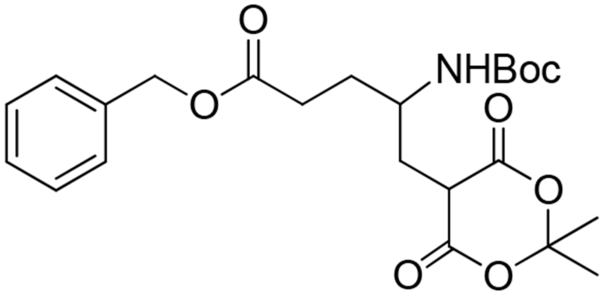

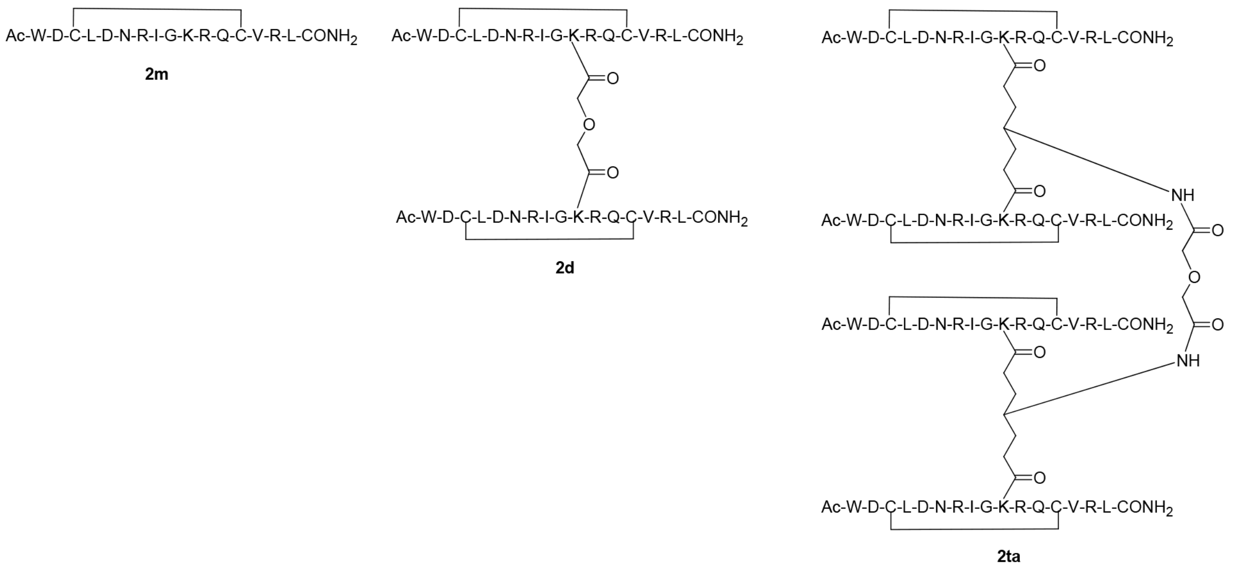

Supplementary Figure S1: Formulae of Compounds 2m, 2d and 2ta used in this work.

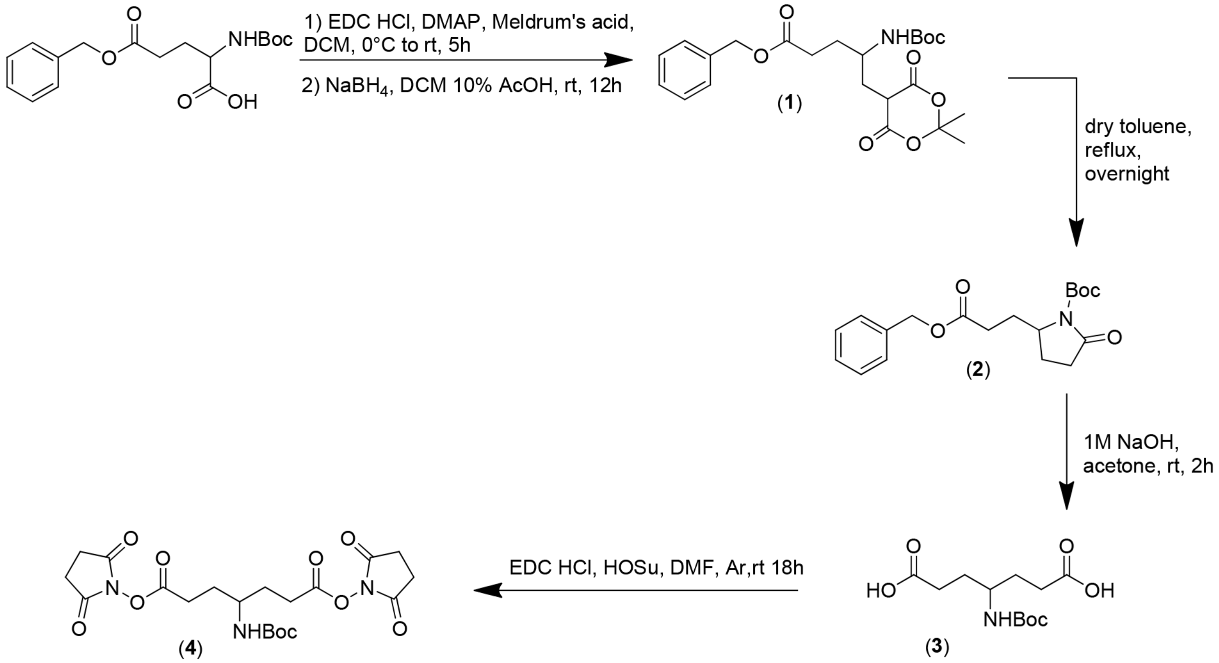

Supplementary Figure S2: Synthetic scheme for the synthesis of dimerisation linker 4.

the solution was diluted with brine, and the organic layer was washed with water, dried over Na_2SO_4, filtered, and evaporated to afford 6.65 g of a crude mixture, which was then purified by precipitation from CH_2Cl_2/diethyl ether to yield 5 g of pure compound **1** (white crystals, yield = 75.1 % calculated for $C_{23}H_{31}NO_8$) : **HPLC** (30-100 % CH_3CN 0.1% TFA, 10 min) rt = 6.8 min; **^{1}H NMR** (300 MHz, DMSO-d_6) : δ = 1.35 (s, 9H, $(CH_3)_3$CCO), 1.66 (s, 3H, C$(CH_3)_3$OCO), 1.76 (s, 3H, C$(CH_3)_3$OCO), 2.00-2.04 (t, ^{3}J = 6.16 Hz, 2H, CH_2CH_2CHNH), 2.33-2.38 (t, ^{3}J = 7.18 Hz, 2H, COCH_2CH_2CH), 3.27-3.58 (m, 2H, CHCH_2CHNH), 3.66-3.81 (m, 1H, CO*CH*CH_2CH), 4.11-4.14 (m, 1H, CH_2*CH*NHCH_2), 5.08 (s, 2H, $C_6H_5CH_2$OCO), 6.64-6.67 (d, ^{3}J = 9.1 Hz, 1H, CHN*H*COO), 7.36 (m, 5H, H_{Ar}).

N-Boc-2-(2-((benzyloxy)carbonyl)ethyl)-5-oxopyrrolidine (2)

A 0.1 M solution of **1** (2 g, 4.44 mmol) in dry toluene was heated under reflux overnight. The solvent was evaporated and the crude mixture dissolved in EtOAc and washed with saturated $NaHCO_3$, brine. and 1N $KHSO_4$. The organic layer was dried over Na_2SO_4 and concentrated *in vacuo*. The crude compound was purified by flash column chromatography (CHex/EtOAc, 3 : 1) to afford pure **2** (800 mg, colorless oil, yield = 54.4 % calculated for $C_{19}H_{25}NO_5$) : **HPLC** (0-100 % CH_3CN 0.1% TFA, 10 min) rt = 6.0 min; **^{1}H NMR** (300 MHz, $CDCl_3$) : δ = 1.54 (s, 9H, $(CH_3)_3$CCO), 1.71-1.94 (m, 2H, CH_2CH_2CHN), 2.13-2.17 (m, 2H, CH_2CH_2CHN), 2.40-2.46 (t, ^{3}J = 7.19 Hz, 2H, NCOCH_2CH_2), 2.48-2.66 (m, 2H, OCOCH_2CH_2), 4.16-4.22 (m, 1H, CH_2*CH*CH_2), 5.15 (s, 2H, $C_6H_5CH_2$OCO), 7.38 (m, 5H, H_{Ar}).

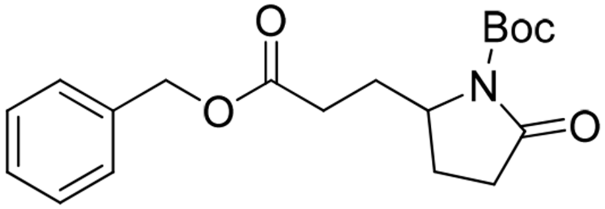

N-Boc-γ(Glu)-OH, N-Boc-4-amino-heptanedioic acid (3)

Compound **2** (800 mg, 2.3 mmol) was dissolved in 4.5 mL of acetone, and 7 mL of 1M aqueous NaOH were added. The reaction was stirred at room temperature for 2 h. Acetone was removed under reduced pressure and the reaction mixture was acidified with 6M HCl to pH = 2. After extraction of the aqueous layer with EtOAc, the combined organic layers were dried over Na_2SO_4 and filtrated. Evaporation of the solvent gave a white solid which was then washed with water and petroleum ether to afford pure compound **3** (320 mg, white solid, yield = 51.8 %, calculated for $C_{12}H_{21}NO_6$) : **HPLC** (0-100 %

CH_3CN 0.1% TFA, 10 min) rt = 5.1 min; **^{1}H NMR** (300 MHz, DMSO-d_6) : δ = 1.37 (s, 9H, $(CH_3)_3CCO$), 1.47-1.64 (m, 4H, CH_2CH_2CHNH), 2.14-2.20 (t, ^3J = 7.78 Hz, 4H, $OCOCH_2CH_2$), 3.33-3.36 (m, 1H, $CH_2CHNHCH_2$), 6.61-6.64 (d, ^3J = 9.0 Hz, 1H, CHN*H*COO), 11.99 (s, 2H, COO*H*).

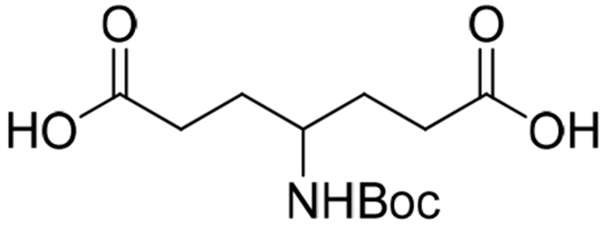

N-Boc-γ(Glu)-OSu (4)

Compound **3** (52 mg, 0.19 mmol) was dissolved in 3 mL of DMF under an Ar atmosphere. After addition of EDC.HCl (137 mg, 0.47 mmol) and HOSu (48 mg, 0.42 mmol), the mixture was stirred at room temperature for 18 h. After completion, the reaction was quenched by addition of 1N $KHSO_4$. The resulting white solid was then filtered, washed with CHex, petroleum ether, followed by diethyl ether, and then dried *in vacuo* to afford 71 mg of crude compound **4** (white solid, yield = 80.0 %) : **HPLC** (0-100 % CH_3CN 0.1% TFA, 10 min) rt = 6.8 min ; **^{1}H NMR** (300 MHz, CD_3CN) : δ = 1.41 (s, 9H, $(CH_3)_3CCO$), 1.72-1.85 (m, 4H, CH_2CH_2CHNH), 2.62-2.67 (t, ^3J = 7.73 Hz, 4H, $OCOCH_2CH_2$), 2.76 (s, 12H, CH_2CH_2CON), 3.58-3.54 (m, 1H, $CH_2CHNHCH_2$), 5.22-5.26 (d, ^3J = 9.09 Hz, 1H, CHN*H*COO) ; **^{13}C NMR** (75 MHz, CD_3CN) δ = 25.9 (CH_2), 26.8 (CH_2), 28.5 (CH_3), 31.2 (CH_2); 45.1 (CH), 79.4 (C), 168.23 (CO), 170.12 (CO) ; **MS** (ESI$^+$) m/z calculated for $C_{20}H_{27}N_3O_{10}$: 469.17 : found $[M + Na]^+$ = 492.1, $[2M + Na]^+$ = 960.7.

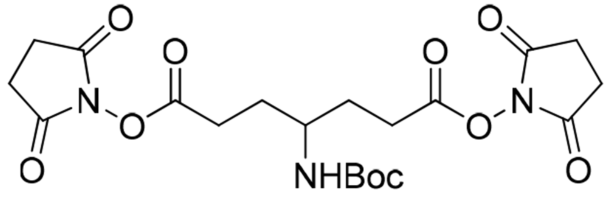

Synthesis of 2m multimers

Synthesis of 2m dimer (5)

The peptide **2m**[1] (40.0 mg, 16 μmol) and the linker (V.13, 3.8 mg, 8.0 μmol) were dissolved in 150 μl of DMF, to obtain a final concentration of 225 mg/ml of M2c. DIEA (5.6 μl, 32.0 μmol) was added to the reaction mixture to reach

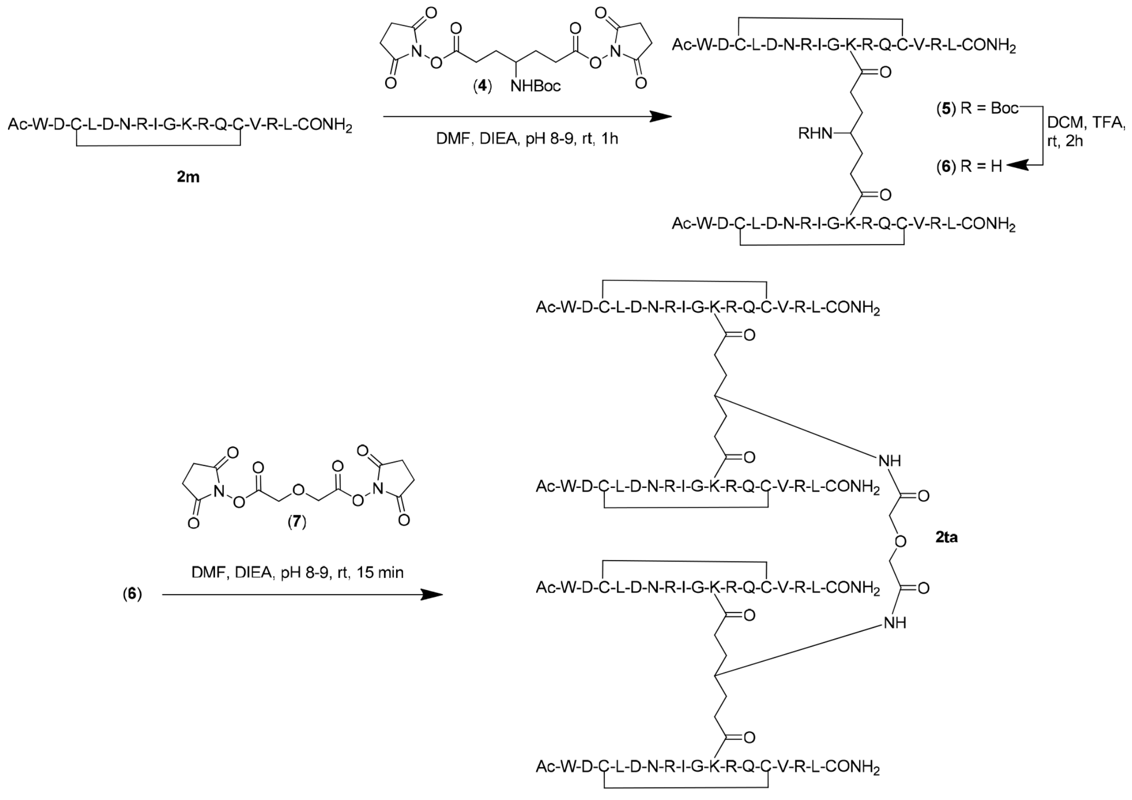

Supplementary Figure S3: Synthetic scheme for the synthesis of 2m tetramer 2ta.

a pH of 8-9. The reaction was stirred at room temperature and followed by HPLC (20%-50% CH_3CN in water, 0.1% TFA, 10 min), and the pH was constantly monitored and maintained basic if necessary by further addition of DIEA. After 2 hours and a total amount of DIEA of 11.2 µl, the reaction was quenched by addition of H_2O until neutral pH, and the crude was purified by semipreparative HPLC (gradient 20% -50% CH_3CN 0.1% TFA, 30 min), to afford the desired compound as a white solid (20.4 mg, 52%). **MW**: 4269.92 (neutral); **Exact mass**: 4267.11; **Analytic HPLC** (20% -50% CH_3CN 0.1% TFA, 10 min) rt 6.0 min, purity > 99%; **ESI-MS (low res)**: *m/z measured* 1423.24 $[M+3H]^{3+}$, 1067.87 $[M+4H]^{4+}$, 854.51 $[M+5H]^{5+}$.

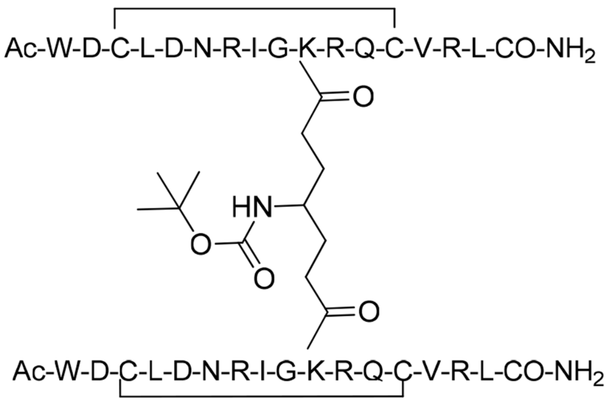

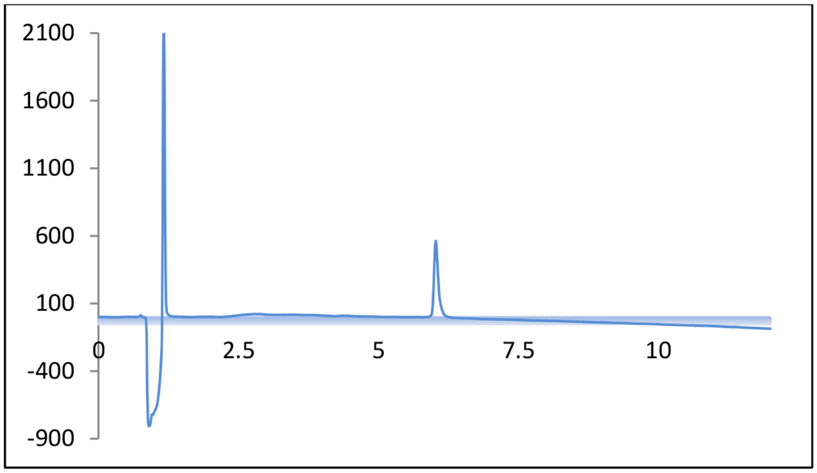

Supplementary Figure S4: RP-HPLC profile of compound 5.

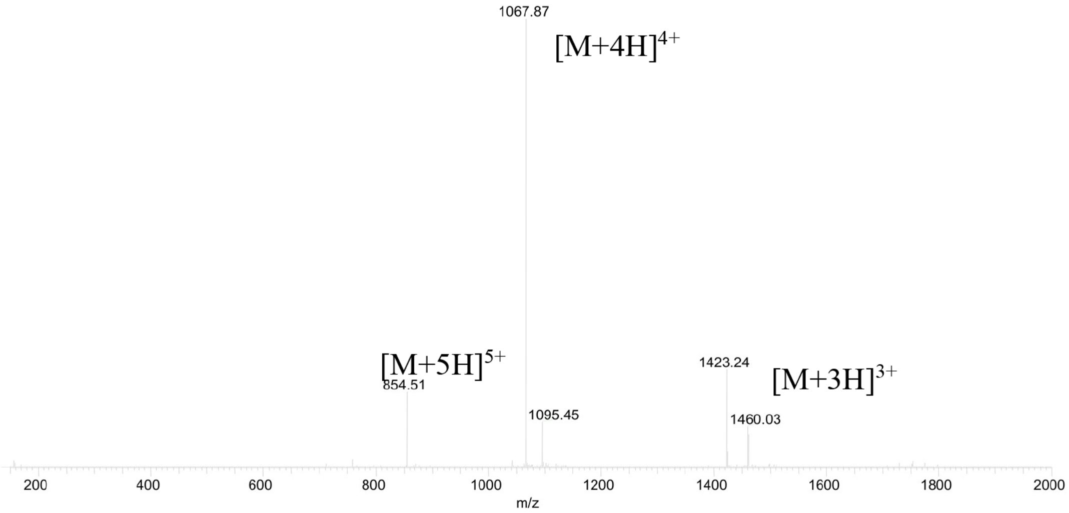

Supplementary Figure S5: ESI-MS spectrum of 5.

Boc deprotection of 2m dimer (6)

The protected dimer (**5**) (15.2 mg, 4.0 µmol) was dissolved in a mixture of 95% TFA-5%DCM (10 ml) and stirred at room temperature monitoring the evolution of the reaction with HPLC. After 2 hours completion was reached, and the solvents were concentrated *in vacuo*. The compound was precipitated as a TFA salt upon diethyl ether addition. The precipitate was isolated by centrifugation, and washed with diethyl ether (x 4). The pure product was obtained as an off white solid (17.4 mg, quantitative). **MW**: 4283.82 (TFA salt); **Exact mass**: 4167.11; **Analytic HPLC**: Rt 5.3 min (20% -50% CH_3CN 0.1% TFA, 10 min), purity 98%; **ESI-MS**: **(low res)**: *m/z measured* 1389.77 $[M+3H]^{3+}$, 1042.87 $[M+4H]^{4+}$, 834.58 $[M+5H]^{5+}$, 695.74 $[M+6H]^{6+}$, 596.52 $[M+7H]^{7+}$.

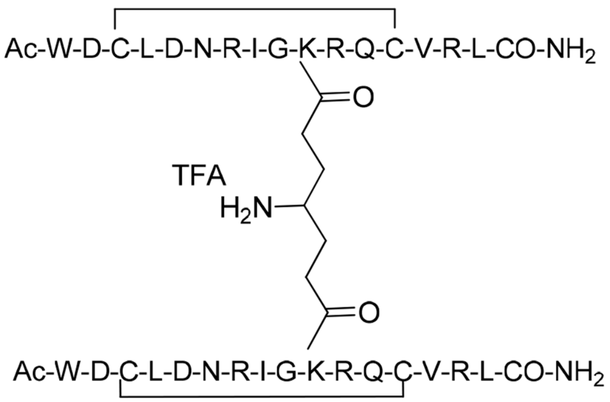

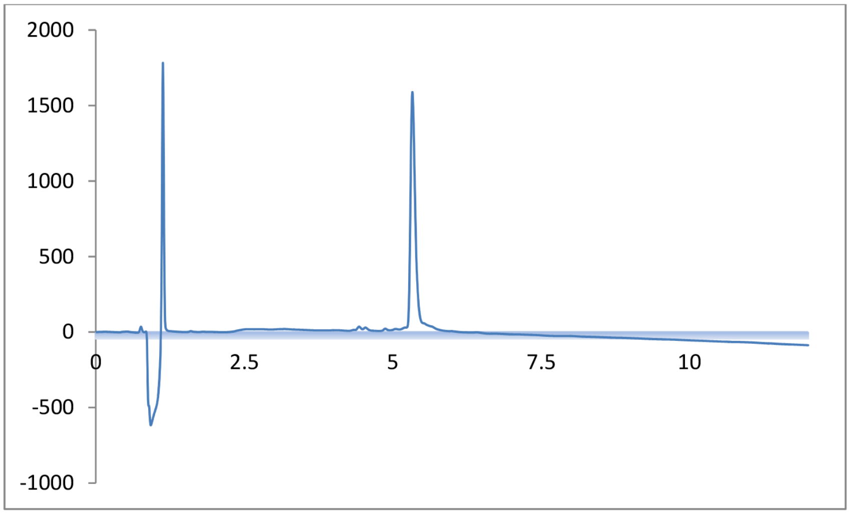

Supplementary Figure S6: RP-HPLC profile of compound 6.

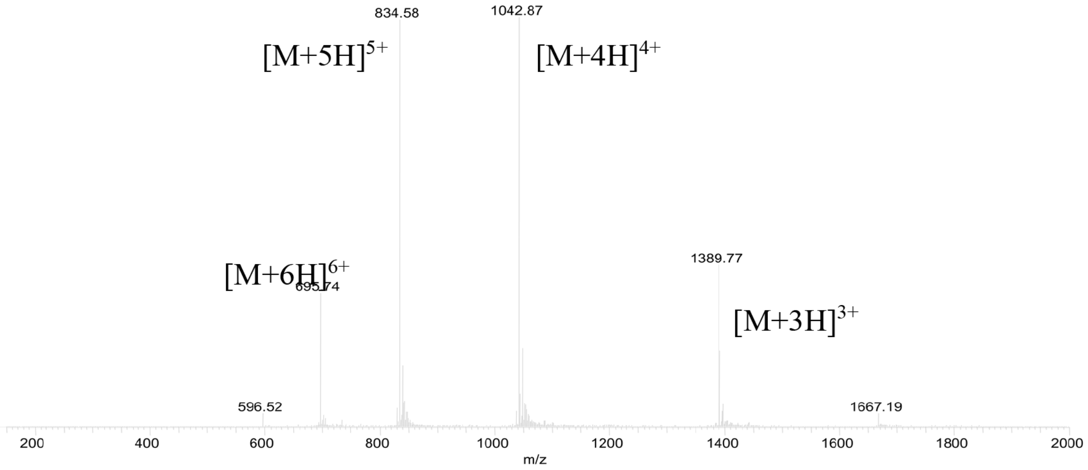

Supplementary Figure S7: ESI-MS spectrum of 6.

Synthesis of 2m tetramer 2ta

2m dimer (**6**) (8.9 mg, 2.0 µmol MW: 4965, considering 7 x TFA salt) and the linker (**7**) (bis(2,5-dioxopyrrolidin-1-yl) 2,2'-oxydiacetate[1], 0.23 mg, 0.7 µmol) were dissolved in 36 µl of DMF, stirring at room temperature. DIEA was subsequently added (1.2 µl, 7.0 µmol) to reach a pH of 9, and the mixture was stirred at room temperature, monitoring the pH. The progression of the reaction was followed by HPLC (20% -50% CH_3CN 0.1% TFA, 10 min, UV-vis detection). After 1.30 hours and an overall addition of 1.7 µl of DIEA, the reaction was quenched by addition of H_2O until neutral pH and the crude was purified with semipreparative HPLC (gradient 20% -50% CH_3CN 0.1% TFA, 30 min, 220 nm), to afford the desired product as a white solid (2 mg, 29%). **MW**: 8437.65 (neutral); **Exact mass**: 8432.1227; **Analytic HPLC** (20% -50% CH_3CN 0.1% TFA, 10 min) rt 5.7 min, purity > 99%; **HRMS**: *m/z measured* 8433.2628 (2109.3157 $[M+4H]^{4+}$, 1687.6564 $[M+5H]^{5+}$, 1406.5500 $[M+5H]^{5+}$).

[1]The synthesis of **2m** and the linker for tetramer formation (compound **7**) are reported in *Cancer Res.* **2010**, 70, 1101.

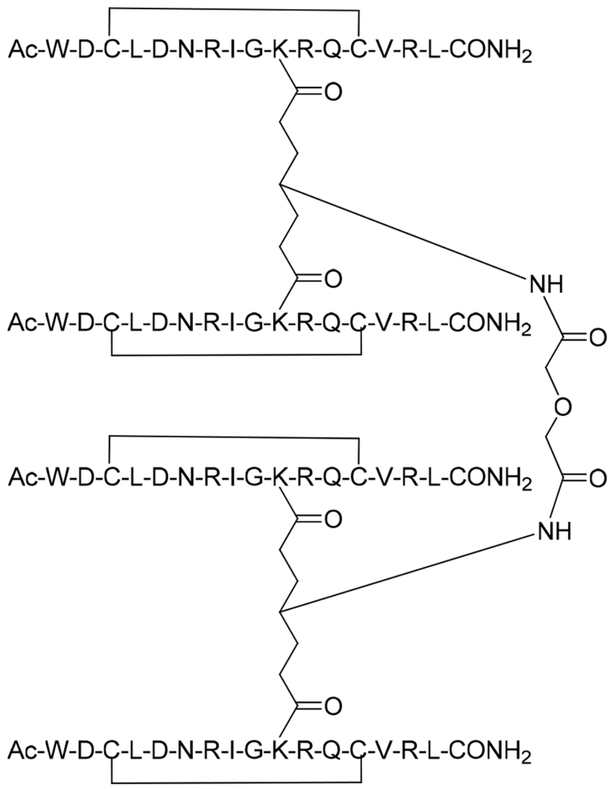

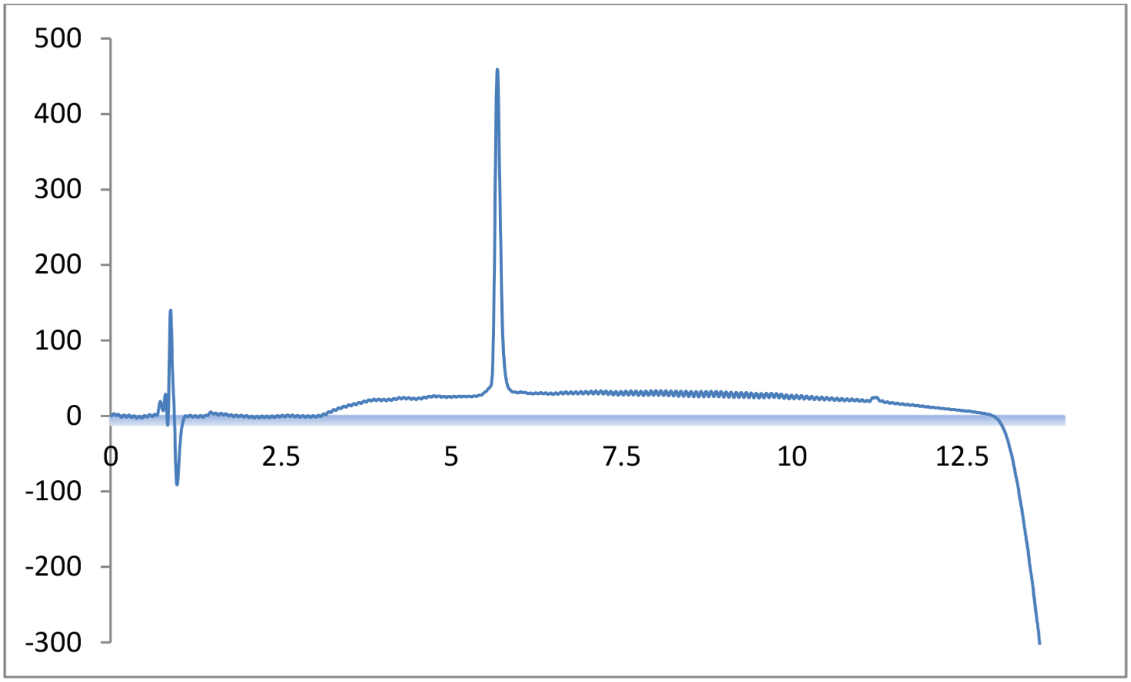

Supplementary Figure S8: RP-HPLC profile of compound 2ta.

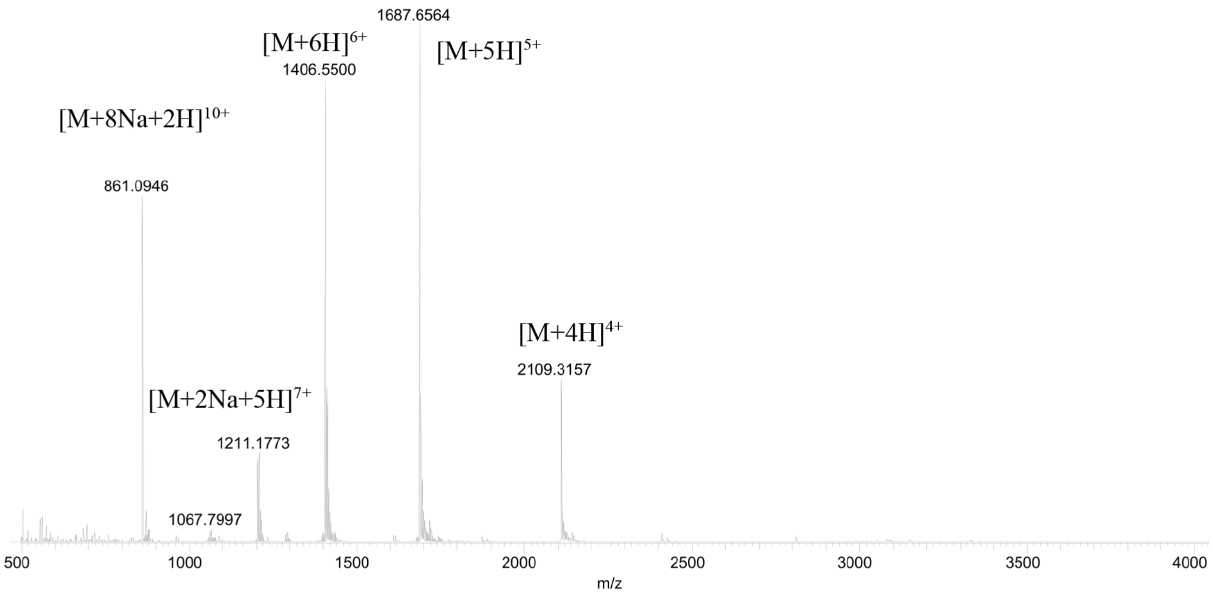

Supplementary Figure S9: ESI-MS spectrum of 2ta.
